# Assessing the Impact of Screen Time on the Motor Development of Children: A Systematic Review

**DOI:** 10.1002/pdi3.70002

**Published:** 2025-04-30

**Authors:** Danyal Bakht, Faiza Yousaf, Zainab Alvi, Muhammad Khan Buhadur Ali, Mirza Muhammad Hadeed Khawar, Luqman Munir, Syed Faqeer Hussain Bokhari, Muhammad Shoaib Qureshi, Mehnahil Raza, Ali Akram Qureshi

**Affiliations:** ^1^ King Edward Medical University Mayo Hospital Lahore Pakistan; ^2^ Services Institute of Medical Sciences Services Hospital Lahore Pakistan

**Keywords:** children, motor development, motor skills, screen time, sedentary behavior

## Abstract

Screen time, defined as the amount of time spent engaging with electronic screens, has become inevitable in modern life. The rise in screen time among children under 5 years old has raised concerns about its association with motor development including gross and fine motor skills. Conflicting evidence on the association of screen time requires more investigation and the planning of targeted interventions. This systematic review aims to explore the relationship between screen time and motor development in children aged 0–7 years, considering various influencing factors like screen type, exposure duration, and context. Following preferred reporting items for systematic reviews and meta‐analyses (PRISMA) and assessing the methodological quality of systematic reviews (AMSTAR) guidelines, a literature search was conducted in May 2024 in databases including PubMed, Cochrane Central Register of Controlled Trials (CENTRAL), and ScienceDirect. Eligible studies were observational or experimental, involved children aged 0–7 years, and assessed the outcomes between screen time and motor development. The quality of the included studies was assessed through Joanna Briggs Institute's (JBI) critical appraisal checklist. Out of 1490 records initially identified, 24 studies met the inclusion criteria for this review. Among these, 17 studies reported a significant negative correlation between screen time and motor development in children, while 5 studies found no statistically significant association. Two studies presented mixed findings, indicating both negative and positive associations between screen time and motor development. Excessive screen time in early childhood is mainly linked to negative effects on motor development. The association varies with screen content and environment, highlighting the need for balanced screen time and early interventions.

## 1 Introduction

In today's digital world, spending time on screens has become a regular part of life. Screen time refers to the hours spent using electronic devices like smartphones, computers, TVs, tablets, and gaming consoles. While it plays an important role in accessing information, education, and staying connected with others, it has also raised concerns. Many studies have looked into how screen time might affect physical and mental health, emphasizing the importance of using it in moderation [1, 2]. The substantial increase in screen time during the past few years, owing partly to the COVID‐19 pandemic, is particularly noteworthy [3]. Especially, an upsurge in screen time among children under 5 years of age has emerged as a recent concern [4, 5, 6]. The negative impact of excessive screen time on children's early development is undeniably a critical area that warrants further exploration.

Early development of children is tracked via developmental milestones, which are considered to be markers of a child's development from infancy through childhood. There is a range of developmental milestones, categorized into five main classes: gross motor, fine motor, language, cognitive, and social‐emotional and behavioural [7]. Achievement of these milestones at the appropriate age is a reliable indicator of normal development. Developmental milestones are tracked between the ages of 6 months and 5 years. Developmental monitoring done by parents and caregivers at home, as well as developmental screening based on validated, research‐based screening tools used by trained healthcare providers, is all essential [8]. The American Academy of Pediatrics (AAP) recommends developmental and behavioral screening for all children during regular well‐child visits at ages of 9, 18, and 30 months [9]. Various screening tools, such as parent‐completed questionnaires and standardized assessments, are combined with the clinical judgment of healthcare professionals to evaluate developmental delays in children [10]. For the assessment for motor development specifically, including gross motor and fine motor skills, a number of screening tools such as Gross Motor Function Measure‐88 (GMFM‐88) and Movement Assessment Battery for Children‐2 (MABC‐2) are available [11, 12].

The critical importance of developmental screening lies in its role in the early detection of developmental delays. Most commonly encountered disorders associated with delayed motor development in children, such as cerebral palsy (CP), muscular dystrophy (MD), autism spectrum disorder (ASD), attention‐deficit/hyperactivity disorder (ADHD), tic disorders etc., have shown better outcomes with early intervention [13, 14, 15]. This highlights the significance of early screening for these disorders, facilitating timely intervention and treatment. Early diagnosis and intervention through developmental screening can result in cost savings over the long term by reducing the need for more intensive interventions or specialized services in later life. Addressing motor developmental delays early can also lead to improved interventional outcomes and increased opportunities for success in school and adulthood [16].

The relation between screen time and the early motor development of children is a complex and multi‐faceted issue that has been a subject of extensive research, especially in recent years. Numerous studies have shown conflicting results. For instance, some researches show a positive correlation between early use of touch screens and development of fine motor skills in toddlers [17]. On the other hand, studies have shown that excessive screen time during early childhood is negatively associated with the development of motor skills [18]. Slowed motor development because of excessive screen time leads to numerous other issues such as delayed language development, impaired social skills, disrupted sleep patterns, and decreased physical activity. The current body of literature presents conflicting and inconclusive findings regarding the relationship between screen time and motor development, with studies varying from degrees of association. This systematic review aims to critically analyze and consolidate existing studies on the impact of screen time on children's motor development. By examining research that investigates the relationship between different types of screen use—such as television, computers, tablets, and smartphones—and the development of motor skills, the review seeks to uncover patterns, trends, and gaps in the literature. The ultimate objective is to provide evidence‐based insights to assist parents, educators, and healthcare professionals in making informed decisions about screen time and its influence on children's physical development.

## 2 Materials and Methods

This systematic review has been reported according to preferred reporting items for systematic reviews and meta‐analyses (PRISMA) and assessing the methodological quality of systematic reviews (AMSTAR) guidelines, ensuring a rigorous and comprehensive evaluation of the included studies.

### 2.1 Search Strategy

In May 2024, a thorough literature search was conducted across multiple electronic databases, including PubMed, Cochrane Central Register of Controlled Trials (CENTRAL), and ScienceDirect. The search strategy was devised collaboratively with a medical librarian and encompassed a blend of pertinent keywords and subject headings pertaining to “screen time,” “sedentary behavior,” “motor development,” “motor skills,” and “children.” For PubMed and Cochrane, the search string employed was (“screen time” OR “digital media” OR “digital devices” OR “electronic media” OR “television” OR “TV” OR “computer” OR “smartphone” OR “tablet use”) AND (“motor development” OR “motor skills” OR “physical development” OR “motor coordination” OR “gross motor skills” OR “fine motor skills” OR “motor abilities”) AND (“children” OR “child” OR “infants” OR “toddlers” OR “preschoolers” OR “youth”). However, due to limitations in using Boolean operators on ScienceDirect, the search strategy was simplified to (“screen time” OR “digital media” OR “digital devices”) AND (“motor development” OR “motor skills” OR “motor abilities”) AND (“children” OR “child” OR “infants”). The search was confined to studies published from the inception of each respective database up to May 2024. Additionally, further studies were unearthed through manual scrutiny of the reference lists of pertinent systematic reviews and included studies.

### 2.2 Eligibility Criteria

Studies were considered eligible for inclusion if they met the following criteria [1]: original research studies with observational (cohort, or case‐control) or experimental (randomized controlled trials, or quasi‐experimental) study designs [2]; study population consisting of children aged 0–7 years [3]; assessment of screen time exposure, defined as time spent viewing/using television, computers, video games, smartphones, or other screen‐based media for recreational purposes [4]; assessment of at least one outcome measure related to motor development or motor skills; and [5] reported quantitative data on the association between screen time and motor development/skills or provided sufficient information to calculate an effect size. Studies were excluded if they met the following criteria [1]: assessed only physical activity or sedentary behavior without specific information on screen time [2]; focused solely on special populations (e.g., children with specific health conditions or disabilities) [3]; case reports, reviews, editorials, or commentaries; or [4] not published in English.

### 2.3 Study Selection

Two independent reviewers screened the titles and abstracts of all retrieved records to identify potentially eligible studies. Full texts were then obtained for all seemingly relevant records and independently assessed by the two reviewers for inclusion based on the eligibility criteria. Disagreements were resolved through discussion and consensus with a third reviewer.

### 2.4 Data Extraction

A standardized form was used to extract data from each included study by one reviewer and checked for accuracy by the other reviewer. Extracted information included study characteristics (author, year, country, and study design), participant details (age, and sample size), screen time exposure details, motor development assessment methods, reported outcomes and conclusions.

## 3 Results

### 3.1 Study Selection Process

**FIGURE 1:**
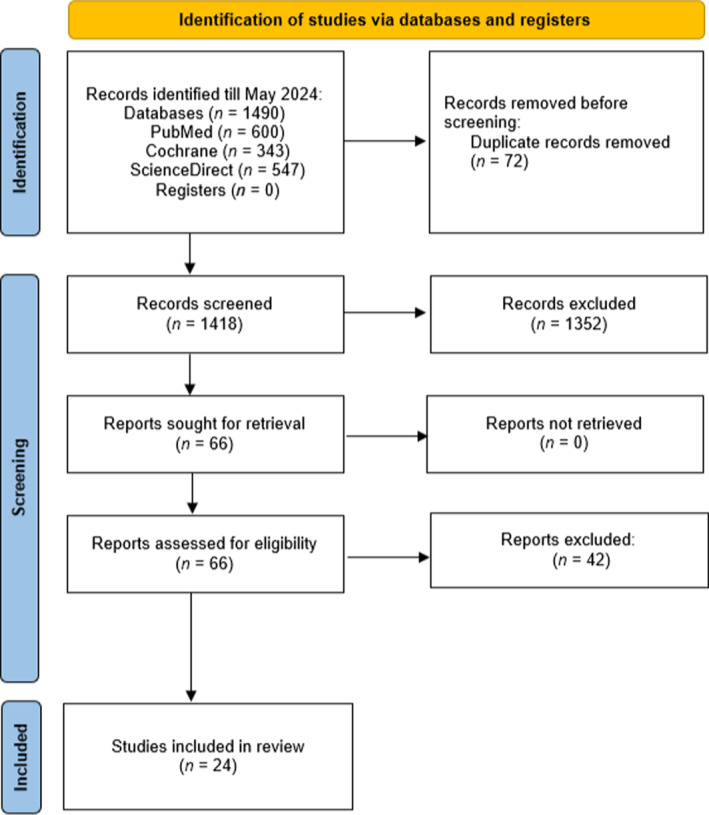
PRISMA diagram showing the study selection process.

The systematic literature searches across all databases initially identified 1490 potentially relevant records. After removing 72 duplicate records, 1418 unique records underwent title and abstract screening by the two independent reviewers. Of these, 66 records were deemed potentially eligible and were retrieved for full‐text review. Ultimately, 24 studies satisfied all of the pre‐specified inclusion criteria and were included in the final systematic review. Common reasons for exclusion at the full‐text stage included: study population not meeting the age criteria, lack of a validated motor development outcome measure, and not assessing or reporting data on screen time exposure. The study selection process is documented using a PRISMA flow diagram, detailing the number of records identified, screened, assessed for eligibility, and included in the final review (Figure 1).

### 3.2 Study Characteristics

The key characteristics of the 24 included studies are summarized in Table 1. These studies originated from 12 different countries across four continents: North America, South America, Europe, and Asia, with the highest number of studies conducted in Brazil (*n* = 5), USA (*n* = 3) and Canada (*n* = 3). The sample sizes ranged considerably across studies, with the smallest study including only 25 participants and the largest as a retrospective cohort study with 129,278 children. Eleven out of the 24 studies had over 500 participants. In terms of study design, there were 9 prospective cohort studies, 11 cross‐sectional studies, 2 retrospective cohort studies, 1 case‐control study, and 1 controlled trial. The studies reviewed encompassed a broad age range from birth to 7 years, enabling a thorough assessment of motor development throughout early childhood. The age groups analyzed included key developmental stages such as 6–60 months, 0–24 months, 24–42 months, and 48–72 months. More specific age intervals were also considered, including 12, 18, 24, 30, and 36 months, and broader ranges such as 35–82 months.

**TABLE 1:** Research characteristics of studies included in this systematic review.

Author	Year of study	Country of study	Type of study	Sample size and composition	Age group
Madigan et al. [19]	2019	Canada	Prospective cohort study	*n* = 2441 *M* = 1622 *F* = 819	24, 36 and 60 months old
Geng et al. [20]	2023	China	Retrospective cohort study	*n* = 129,278 *M* = 67,780 *F* = 61,498	3.950 years old
Suggate et al. [21]	2021	Germany	Prospective cohort study	*n* = 117 *M* = 62 *F* = 55	57.76 months old at the beginning and 79.86 months old at the end of study
de‐andrade Leão et al. [22]	2023	Brazil	Prospective cohort study	*n* = 8506 Cohort = 4231 (2004 born) Cohort = 4275 (2005 born)	4 years old
Nobre et al. [23]	2021	Brazil	Cross‐sectional study	*n* = 180 *M* = 84 *F* = 96	Between 24 and 42 months old
Putnick et al. [24]	2023	USA	Prospective cohort study	*n* = 3894 (M)	12, 18, 24, 30 and 36 months old
Lin et al. [25]	2017	Taiwan region, China	Quasi experimental study	*n* = 40 *M* = 26 *F* = 14	48–72 months old
Felix et al. [26]	2020	Brazil	Cross‐sectional study	*n* = 926 *M* = 475 *F* = 451	4–6 years old
Binet et al. [27]	2024	Canada	Prospective cohort study	*n* = 315 *M* = 171 *F* = 144	3.5 and 4.5 years old
Varadarajan et al. [28]	2021	India	Cross‐sectional study	*n* = 718 *M* = 358 *F* = 360	6 months to 5 years old
Dadson et al. [29]	2020	Australia	Cross‐sectional study	*n* = 25 *M* = 9 *F* = 16	4–7 years old
Martzog et al. [30]	2022	Germany	Retrospective cohort study	*n* = 141 *M* = 71 *F* = 70	35–82 months old
Rocha et al. [31]	2021	Brazil	Cross‐sectional study	*n* = 3155 *M* = 1582 *F* = 1573	0–60 months old
Takahashi et al. [32]	2023	Japan	Prospective cohort study	*n* = 7097 *M* = 3674 *F* = 3423	2–4 years old
Chaibal and Chaiyakul [33]	2022	Thailand	Cross‐sectional study	*n* = 85 *M* = 43 *F* = 42	2–5 years old
Daud et al. [34]	2020	Malaysia	Case‐control study	*n* = 128 *M* = 64 *F* = 64	5–6 years old
Yuan et al. [35]	2024	China	Cross‐sectional study	*n* = 817 *M* = 441 *F* = 376	3–6 years old
McArthur et al. [36]	2020	Canada	Prospective cohort study	*n* = 1910 *M* = 996 *F* = 914	24–60 months old
Operto et al. [37]	2023	Switzerland	Cross‐sectional study	*n* = 185 *M* = 105 *F* = 80	55 months old
Schmidt et al. [38]	2009	USA	Prospective cohort study	*n* = 872 *M* = 433 *F* = 439	0–2 years old
Webster et al. [18]	2019	USA	Prospective cohort study	*n* = 126 *M* = 58 *F* = 68	Average = 3.4 years old *M* = 3.4 years old *F* = 3.3 years old
Lucena Martins et al. [39]	2020	Brazil	Cross‐sectional study	*n* = 212 For the 3‐year‐old group *M* = 39 *F* = 35 For the 4‐year‐old group *M* = 33 *F* = 40 For the 5‐year‐old group *M* = 37 *F* = 28	For the 3‐year‐old group *M* = 3.58 years old *F* = 3.67 years old For the 4‐year‐old group *M* = 4.12 years old *F* = 4.25 years old For the 5‐year‐old group *M* = 5.01 years old *F* = 5.45 years old
Moon et al. [17]	2019	South Korea	Cross‐sectional study	*n* = 117 For the 3‐year‐old group *M* = 22 *F* = 18 For the 4‐year‐old group *M* = 19 *F* = 17 For the 5‐year‐old group *M* = 22 *F* = 19	For the 3‐year‐old group aged averagely 3.5 years For the 4‐year‐old group aged averagely 4.4 years For the 5‐year‐old group aged averagely 5.4 years
Lin et al. [40]	2015	Taiwan region, China	Cross‐sectional study	*n* = 150, Exposure group: *M* = 54 and *F* = 21. Control group: *M* = 54 and *F* = 21.	12–35 months old

Abbreviations: F, Female; M, Male; *n*, Total number.

### 3.3 Study Summary

The studies investigated the association of screen time and motor development in children, considering diverse durations and types of screen exposure. Screen time durations ranged from as low as less than 30 min to as high as 25 h per week, with variations observed across different age groups. Various screen types were examined, including TV, smartphone, tablet, computer, and gaming system. Exposure durations varied widely, with some studies focusing on daily exposure while others assessed weekly or monthly averages. Motor development was assessed using a range of standardized scales such as the Ages and Stages Questionnaire‐3 (ASQ‐3), Movement Assessment Battery for Children‐2, Bayley‐3, and the Test of Gross Motor Development‐2/3 (TGMD‐2 and TGMD‐3) etc. Results from various studies suggest a complex relationship between screen time and motor skills development in children. While most studies have found associations between excessive screen exposure and lower developmental scores, suspected developmental coordination disorder (DCD), and reduced motor performance, particularly in fine motor skills (FMS) and gross motor skills (GMS), few studies have shown minimal or no significant associations between screen time and motor skills. In total, 24 studies were included in the final analysis. Of these, 17 studies demonstrated a negative association between screen time and motor development, 5 studies demonstrated no significant association, and 2 studies reported both negative and positive associations. Table 2 contains a tabulated overview of the individual study findings.

**TABLE 2:** Summary of the main findings of studies included in this systematic review.

Author	Screen time duration	Type of screen used	Variable studied	Assessment method	Outcomes/Results	Conclusion
Madigan et al. [19]	17 h/w at 24 months, 25 h/w at 36 months, 11 h/w at 60 months.	TV, and computer	GMS and FMS	ASQ‐3	More screen time at 24 months linked to lower developmental scores at 36 months; the same trend was observed at 36 months for scores at 60 months.	Negative association
Geng et al. [20]	1–2 h/d	TV, smartphone, computer, and tablet	GMS, FMS, and balance	LDCDQ	Excessive screen exposure is linked to a higher risk of suspected DCD and reduced motor performance, including GMS and FMS as well as general coordination.	Negative association
Suggate et al. [21]	From less than 30 min to 5 h/d	TV, computer, tablet, playing consoles and smartphones	FMS	MABC	Screen time is negatively associated with FMS in children.	Negative association
de‐andrade Leão et al. [22]	3.4 h/d (2004 born) 4.4 h/d (2005 born)	TV, video game, computer, tablet and smartphone	FMS and GMS	BDI	Minimal association between screen time and child neurodevelopment was observed.	No significant association
Nobre et al. [23]	2 or < 2 h/d	TV, tablet and smartphone	FMS, GMS and language skills	Bayley‐3	Screen time is positively associated with language development, while no significant association with FMS or GMS.	No significant association
Putnick et al. [24]	At 12 months: 1 h/d. At 30 months: 2 h/d.	TV and computer	FMS and GMS	ASQ‐3	The decreased chance of toddlers peer play time is associated with motor developmental delay.	Negative association
Lin et al. [25]	60 min/w for 1 month old	Smartphone, iPad and tablet	Fine motor precision, fine motor integration and manual dexterity	BOT‐2	Motor development improved in children who did not used touch screen tablet over 24 weeks.	Negative association
Felix et al. [26]	2 h/d	TV, video game, tablet and smartphone	FMS and GMS	GMQ	Excessive screen media use increased the risk of a low GMQ by 72%.	Negative association
Binet et al. [27]	3.5 h/d	TV, computer, playing console, iPad, tablet, leapPad, iTouch and smartphone	FMS and GMS	ASQ‐3	Higher levels of preschooler screen time are associated with a greater risk of experiencing delays in the achievement of developmental milestones of motor domain 1 year later.	Negative association
Varadarajan et al. [28]	2.39 h/d	Smartphone and TV	FMS and GMS	Communication DEALL developmental checklist	Children with screen time more than 1 h showed developmental delays in the motor domain.	Negative association
Dadson et al. [29]	563.3 min/w	Smartphone and tablet	FMS, inhand manipulation, visual motor integration skills	BOT‐2, TIHM‐R, PPEDC, BBDT‐6 of visual‐motor integration and SPM—home form	Statistically significant negative correlations between children's screen‐time, VMI, fine motor skills, IHM, SP and enjoyment of play.	Negative association
Martzog et al. [30]	Media: 1.51 h/d. TV: 0.92 h/d.	TV, smartphone and tablet	FMS	MABC	Greater media use at a young age correlates with lower fine motor skill development in early childhood.	Negative association
Rocha et al. [31]	TV: 1.5 h/d, touch devices: 0.6 h/d. Video game: 0.3 h/d. Total exposure: 2.4 h/d.	TV, smartphone and tablet	FMS and GMS	ASQ‐3	There was no significant association between screen time and FMS and GMS.	No significant association
Takahashi et al. [32]	3440 children had less than 1h. 2095 had 1h to less than 2h. 1272 had 2h to less than 4h, and 290 had more than 4h.	TV, mobile phone and tablet	FMS and GMS	ASQ‐3	More media usage relates negatively to FMS development in early childhood.	Negative association
Chaibal and Chaiyakul. [33]	2–3 years old group: 115.62 min/d, 3–4 years old group: 84.82 min/d, 4–5 years old group: 75.48 min/d.	Smartphone and tablet	Gross motor, fine motor‐adaptive, personal‐social and language skills	Denver II assessment	A significant correlation between SP/Tb usage duration and gross motor development (*p* = 0.036). No significant correlation observed for other variables.	Negative association
Daud et al. [34]	HUTSTG > 120 min/d. LUTSTG > 120 min/d.	Phone and tablet	Hand skills	CHSQ, ACHS	Children in the LUTSTG group had better hand skills in all domains of CHSQ and ACHS, compared to the children in HUTSTG group.	Negative association
Yuan et al. [35]	3 years old: 1.17 h/d, 4 years old: 1.39 h/d, 5 years old: 1.58 h/d, 6 years old: 1.66 h/d.	TV, cellphone and computer	Gross motor development	TGMD‐3	A significant negative correlation between gross motor development and screen time.	Negative association
McArthur et al. [36]	24 months old: 2.33 h/d, 36 months old: 3.47 h/d, 60 months old: 1.53 h/d	TV, computer, gaming system, and other screen‐based devices	Behaviour and developmental milestones	BASC, ASQ‐3	Consistent high screen usage predicts lower total developmental milestone achievement scores and increased behavioral issues.	Negative association
Operto et al. [37]	SP = 0.88 h, Tb = 0.43 h, PC = 0.14 h, VG = 0.06 h, and TV = 1.57 h	Smartphone, tablet, personal computer, video games and TV	FMS	APCM‐2	There was no significant association between screen time and FMS.	No significant association
Schmidt et al. [38]	average = 1.2 h/d, 0.9 h/d at 6 months old, 1.2 h/d at 1 year old and 1.4 h/d at 2 years old	TV	Language and visual motor skills	PPVT‐III for vocabulary, WRAVMA for visual motor skills	Television viewing is not associated with the language and visual motor skills.	No significant association
Webster et al. [18]	Average = 5.1 h/d, TV = 1.9 h/d, SP = 0.9 h/d, VG = 0.6 h/d, Tb = 1.2 h/d, and computer = 0.8 h/d	TV, smartphone, video game, tablet and computer	FMS and GMS, manual dexterity, balance, and aiming and catching	TGMD‐3 MABC‐2	Children's motor skills were inversely related to screen‐time.	Negative association
Lucena Martins et al. [39]	For 3 years old, M: 173.94 min/d F: 170.26 min/d For 4 years old, M: 172.34 min/d F: 146.62 min/d For 5 years old, M: 189.67 min/d F: 149.8 min/d	TV, smartphone tablet and computer	Fundamental motor skills, which include GMS such as locomotor and ball Skills.	TGMD‐2	For 3‐year‐old children, there was a weak positive relationship between screen time and locomotion and object control skills. In 4‐ and 5‐year‐old children, the relationships became negative, with a stronger negative association in 5‐year‐old children.	Positive association at 3 years old. Negative association at 4–5 years old.
Moon et al. [17]	For 3 years old < 1 h For 4 years old 1 to less than 2 h For 5 years old 2 to less than 3 h	Smartphone, tablet and computer	FMS and expressive language	The Korean developmental screening test	Smart device use was negatively associated with social and language skills but positively associated with fine motor skills	Both negative and positive association
Lin et al. [40]	Exposure: > 2 h/d (average 137.2 min/d) Control: < 2 h/d (average 16.3 min/d)	TV	FMS and GMS	BSID‐II and PDMS‐2	Language and motor delays in young children were significantly associated with the amount of time spent on television.	Negative association

*Note:* Table 2 provides an overview of the key findings from the included studies.

Abbreviations: APCM‐2, Abilità Prassiche e Della Coordinazione Motoria—2a Edizione; ASQ‐3, Ages and Stages Questionnaire; BASC, Behaviour Assessment System for Children; BBDT‐6, Berry Buktenica Developmental Test Sixth Edition; BDI, Battelle Development Inventory; BOT‐2, Bruininks‐Oseretsky Test for Motor Proficiency; TIHM‐R, Test of In‐Hand Manipulation—Revised; PPEDC, Pretend Play Enjoyment Developmental Checklist; BSID‐II, Bayley Scales of Infant Development‐Second Edition; F, Female; FMS, Fine Motor Skills; GMS, Gross Motor Skills; HUTSTG, High Usage Touch‐Screen Technology; IHM, In‐Hand Manipulation; LDCDQ, Little Developmental Coordination Disorder Questionnaire; M, Male; MABC‐2, Movement Assessment Battery for Children‐Second Edition; PDMS‐2, Peabody Developmental Motor Scales‐Second Edition; PPVT‐III, Peabody Picture Vocabulary Test–Third Edition; SP, Sensory Processing; SPM, Sensory Processing Measure; TGMD‐2/3, Test of Gross Motor Development‐2/3 Edition; VMI, Visual‐Motor Integration; WRAVMA, Wide Range Assessment of Visual Motor Abilities.

### 3.4 Quality Assessment

The studies that fit the eligibility criteria included 11 cross‐sectional studies, 11 cohort studies, 1 case‐control study and 1 quasi experimental study. The quality of these studies was assessed using the Joanna Briggs Institute (JBI) critical appraisal checklist. Among the 11 cross‐sectional studies, 6 were of high quality and 5 were of medium quality. Among the 11 cohort studies, 4 were of high quality, 5 were of medium quality and 2 were of low quality. And the included case‐control study and quasi experimental study were of high quality. The details of Quality Assessment are given in the Table 3.

**TABLE 3:** JBI critical appraisal of studies.

Sr No	Author name	Study‐type	JBI appraisal score	Quality assessment	Percentage
1	Nobre et al., 2021 [23]	Cross‐sectional	7/8	High	87.5%
2	Felix et al., 2020 [26]	Cross‐sectional	8/8	High	100.0%
3	Varadarajan et al., 2021 [28]	Cross‐sectional	7/8	High	87.5%
4	Dadson et al., 2020 [29]	Cross‐sectional	7/8	High	87.5%
5	Rocha et al., 2021 [31]	Cross‐sectional	6/8	High	75.0%
6	Chaibal and Chaiyakul, 2022 [33]	Cross‐sectional	5/8	Medium	62.5%
7	Yuan et al., 2024 [35]	Cross‐sectional	7/8	High	87.5%
8	Operto et al., 2023 [37]	Cross‐sectional	6/8	High	75.0%
9	Lucena martins et al., 2020 [39]	Cross‐sectional	4/8	Medium	50.0%
10	Moon et al., 2019 [17]	Cross‐sectional	4/8	Medium	50.0%
11	Lin et al., 2015 [40]	Cross‐sectional	4/8	Medium	50.0%
12	Madigan et al., 2019 [19]	Prospective cohort study	8/11	High	72.7%
13	Geng et al., 2023 [20]	Retrospective cohort study	8/11	High	72.7%
14	Suggate et al., 2021 [21]	Prospective cohort study	6/11	Medium	54.5%
15	De‐andrade Leão et al., 2023 [22]	Prospective cohort study	8/11	High	72.7%
16	Putnick et al., 2023 [24]	Prospective cohort study	6/11	Medium	54.5%
17	Binet et al., 2024 [27]	Prospective cohort study	7/11	Medium	63.6%
18	Martzog et al., 2021 [30]	Retrospective cohort study	7/11	Medium	63.6%
19	Takahashi et al., 2023 [32]	Prospective cohort study	7/11	Medium	63.6%
20	McArthur et al., 2020 [36]	Prospective cohort study	5/11	Low	45.5%
21	Schmidt et al., 2009 [38]	Prospective cohort study	8/11	High	72.7%
22	Webster et al., 2019 [18]	Prospective cohort study	5/11	Low	45.5%
23	Daud et al., 2020^34^]	Case‐control	7/10	High	70.0%
24	Lin et al., 2017 [25]	Quasi experimental study	7/9	High	77.8%

## 4 Discussion

The link between screen time and children's motor development has gained considerable attention in recent years, driven by growing concerns about the widespread use of digital media in daily life. This systematic review compiles and analyzes findings from various studies to clarify the complex relationship between screen time and motor development in children, highlighting both the potential risks and benefits.

Literature indicates that excessive screen time presents a range of risks to children's health and development. Previous research has linked prolonged screen exposure to several negative outcomes. In addition to contributing to poor posture and scoliosis, extended screen use has been associated with impairments in motor coordination, delays in visual‐perceptual development, and adverse effects on cognitive development. Moreover, it may lead to issues like poor concentration, reduced creativity, and deviations in behavior [41]. The study by Geng et al. highlighted a concerning association between prolonged screen time and an increased risk of DCD [20]. Additionally, heightened screen exposure has been associated with diminished microstructural integrity of brain white matter tracts in preschool‐aged children, underscoring the potential neurological impact of excessive screen use on young minds [42]. These findings emphasize the importance of monitoring and limiting screen time in children to reduce the potential adverse effects on their physical and cognitive development.

Several studies have established a clear inverse relationship between higher levels of screen time and poorer performance in motor development. For example, Geng et al. conducted a large retrospective cohort study involving over 129,000 children in China, which revealed that excessive screen exposure was linked to a higher risk of suspected DCD and reduced motor performance, including gross and fine motor skills, as well as general coordination [20]. Similarly, a prospective cohort study by Madigan et al. found that greater screen time at 24 months was associated with lower developmental scores, including motor domains, at 36 and 60 months [19]. Additional studies of Felix et al., Binet et al., Varadarajan et al., and Yuan et al. consistently reported negative association of prolonged screen time with various aspects of motor development, such as general motor quotient, fundamental movement skills, and overall gross motor development [26, 27, 28, 35]. This negative association is further supported by research indicating that excessive and inopportune digital media use can have adverse effects on child development. Putnick et al. suggested that one possible mechanism for the association between screen time and motor development is the displacement of time spent playing with peers. The study found that children who engaged in more screen time from 12 to 36 months spent less time playing with peers during the same period. This reduced peer interaction was associated with higher odds of developmental delays in four of five developmental domains [24]. The sedentary electronic lifestyle prevalent among young children could also potentially account for the observed negative correlation between FMS and screen time [39]. Additionally, studies have found that more screen time is inversely related to less ideal FMS competence, with children who engage in more screen time performing worse on the MABC‐2 manual dexterity subscale. Manual dexterity, critical for activities such as drawing, writing, and academic achievement, suffers significantly as children exceed recommended screen time, leading to poor skills development [18]. Conversely, some studies have reported no significant association between screen time and motor development outcomes. Rocha et al.'s cross‐sectional study found no correlation between screen time and FMS or GMS assessed using the ASQ‐3 [31]. Similarly, Operto et al. observed no significant relationship between screen time and fine motor skills evaluated by the Assessment of Motor and Process Skills (AMPS‐2) in preschoolers [37]. These contradictory findings may be explained by differences in the types and durations of screen activities, age‐related developmental factors that influence outcomes, inconsistencies in research methods and analysis, and variations in sample characteristics such as socioeconomic status and cultural backgrounds. Additionally, factors like parental involvement, the quality of screen content, and individual differences in children's susceptibility to the effects of screen time could also play a role. Interestingly, some research suggests potential benefits of moderate screen time exposure, particularly for fine motor skills. Moon et al.'s cross‐sectional study reported that smart device use was positively associated with fine motor skills in preschoolers, despite being negatively associated with social and language skills [17]. These findings may be linked to the potential role of interactive touchscreen devices in enhancing fine motor skill development. Such devices often engage children in activities that require hand‐eye coordination, dexterity, and manual manipulation, which could positively influence motor skills.

The context and content of screen time are critical factors to consider when interpreting these findings. The association of screen media quality with motor development remains a topic of debate. In an experimental study, researchers compared fine motor skill development in children who used tablets with those engaged in real‐world fine motor activities. The study showed that children who did not use tablets showed better fine motor skills compared to tablet users [43]. However, studies such as that conducted by Moon et al. revealed contrasting results, suggesting a positive correlation between tablet use and improved fine motor skills [17]. Conversely, the study of Nobre et al. found no significant association between the quality of interactive media use and gross motor development, suggesting that the relationship between screen media quality and motor development may vary depending on the specific type of activity [44].

The age of the child significantly influences the association between screen time and motor development, with studies revealing age‐specific patterns. For instance, Lucena Martins et al. noted a weak positive relationship between screen time and locomotor and object control skills in 3‐year‐old children, which turned negative in 4‐ and 5‐year‐old children, with stronger negative associations observed in the older age group [39]. Research indicates that children aged three to six often lack time for physical activities, and the prevalence of electronic devices like TV and cell phone limits their opportunities for physical engagement, presenting a dilemma in their learning and motor development journey [45, 46].

It is important to acknowledge the limitations of the included studies and interpret the findings with caution. Limitations of this systematic review encompass the heterogeneous study designs, ranging from prospective cohort studies to cross‐sectional analyses, leading to potential methodological discrepancies. Variability in sample sizes and compositions, diverse assessment methods for screen time exposure and motor development, and the potential influence of unmeasured confounders like socioeconomic status pose challenges to the generalizability of findings. Another limitation is the reliance on just three electronic databases—PubMed, CENTRAL, and ScienceDirect—for the literature search. Although we are confident that we have captured the majority of relevant articles, there remains a possibility that some important studies may have been overlooked. Additionally, the review may be subject to publication bias, given the tendency for significant results to be published. Limited longitudinal data and the absence of comprehensive assessment of screen media quality further constrain the ability to establish causal relationships between screen time and motor development.

The overall evidence indicates that excessive screen time may negatively impact motor development in children, particularly in relation to GMS and overall motor coordination. However, the relationship between screen time and fine motor skills seems more complex, with some studies suggesting potential benefits from moderate, interactive screen time exposure. Future research should aim to clarify the specific mechanisms behind these associations, taking into account of factors such as the content and context of screen time, age‐related differences, and potential mediators or moderators.

## 5 Conclusion

This systematic review offers a thorough examination of the relationship between screen time and motor development in children aged 0–7 years. Analyzing 24 studies, it reveals a generally negative correlation between excessive screen time and motor development, with particular concerns regarding both fine and gross motor skills. However, the association is complex, with the nature and extent of the relationship varying depending on the content and context of screen use. The findings suggest that prolonged screen exposure, especially beyond recommended limits, is largely associated with delays in motor skill development. Many studies indicate that children who spend more time on screens tend to score lower on motor development assessments, such as the ASQ‐3 and MABC‐2. This correlation is especially evident when screen time replaces activities that support physical and motor development, such as active play and interactive engagement. Nevertheless, not all studies included in the review show this negative trend. A few report minimal or no significant impact of screen time on motor development, suggesting that factors like the type of content consumed and parental involvement may help reduce some of the negative effects.

## Author Contributions

In this systematic review, D.B. (Corresponding Author) led the project, managing the overall research process, including the conceptualization, development of the methodology, and manuscript preparation. F.Y. made substantial contributions to data collection and analysis, ensuring the integrity and precision of the collected data. Z.A. performed an extensive literature search and assisted in drafting the initial manuscript. M.K.B.A. contributed to the data analysis and the interpretation of the findings. M.M.H.K. provided essential insights and assisted in refining the methodology. L.M. played a key role in reviewing and editing the manuscript, ensuring its clarity and coherence. S.F.H.B. supported the project by critically revising the manuscript for significant intellectual content. M.S.Q. aided in data validation and final proofreading. M.R. was involved in the literature search and provided constructive feedback on the manuscript drafts. Finally, A.A.Q. managed the formatting and submission process, ensuring compliance with the journal's requirements.

## Disclosure

During the preparation of this work, the authors used CHATGPT 3.5 in order to refine language and improve text structure. After using this tool/service, the authors reviewed and edited the content as needed and takes full responsibility for the content of the publication.

## Ethics Statement

In accordance with the systematic review framework, it is important to highlight that formal ethical clearance was deemed unnecessary, as per the discretionary exemption granted by the regulatory authorities at King Edward Medical University.

## Consent

The authors have nothing to report.

## Conflicts of Interest

The authors declare no conflicts of interest.

## Data Availability

All data generated or analyzed during this study are fully included within this publication. For any further information or clarification, please contact the corresponding author.
